# Effect of autologous platelet-rich fibrin on implant stability and crestal bone levels in the maxilla - An observational study

**DOI:** 10.6026/973206300221824

**Published:** 2026-03-31

**Authors:** Shalu Sara Aby, Byju Paul Kurian, Litto Manual, Fathima Mohamad Najeeb, Asha Mathew, Krishnendhu Sai, Sandra S Anand

**Affiliations:** 1 Department of Prosthodontics, Mar Baselios Dental College, Kothamangalam, Kerala, India; 2 Consultant Prosthodontist, Thrikkakara Municipal Co-operative Hospital, Kakkanad, Kerala, India

**Keywords:** Platelet-rich fibrin, crestal bone loss, implant stability quotient, D3 bone, dental implants

## Abstract

Early implant stability and crestal bone preservation remain critical challenges for successful maxillary implant placement in D3 bone density, requiring effective adjunctive therapies. This study compared 30 maxillary implants across PRF-assisted and control groups, evaluating implant stability quotient (ISQ) values and crestal bone alterations over two months post-placement. The PRF group demonstrated significantly higher ISQ values and minimal crestal bone loss compared to controls (p < 0.05), indicating enhanced early osseointegration. PRF effectively supported implant primary stability while preserving surrounding bone architecture during the critical initial healing phase. These findings establish PRF as a valuable adjunctive therapy for optimizing early outcomes in challenging maxillary D3 bone conditions.

## Background:

The dental implants have revolutionized the field of dentistry, with success rates of 90-95% and serve as an alternative to conventional prostheses by providing fixed, functional, and esthetically acceptable restorations. Rehabilitation of edentulous jaws through dental implant placement not only prevents injury to adjacent teeth but also enables the patient to attain and sustain function over an extended period [1]. Branemark defined "osseointegration" as "a direct structural and functional connection between ordered, living bone and the surface of a load-carrying implant" in 1985 [2]. Successful osseointegration relies on several techniques and patient-specific circumstances, with implant stability, which includes two primary stability and secondary stability being the clinical indicators. The primary stability is the mechanical connection of an implant with the adjacent bone, whereas the secondary or biological stability is determined by bone regeneration and remodelling processes [3]. The absence of clinical mobility or excellent stability is a critical factor in implant success and a criterion for implant anchorage quality in bone [4]. Inadequate stability or excessive micro-movement during early healing may impair osseointegration, leading to fibrous encapsulation; hence, enhancing early biological regeneration and preserving crestal bone are particularly critical in low-density maxillary bone. The application of bone morphogenetic proteins or cell adhesion molecules to the implant surface helps the osteoblasts to grow, enhancing healing and improving functional integration [5]. Autologous platelet-rich fibrin (PRF) is a second-generation platelet concentrate [6]. PRF is obtained from the patient's blood without anticoagulants and consists of a dense fibrin matrix enriched with platelets, leukocytes, cytokines, and growth factors, including platelet-derived growth factor (PDGF), transforming growth factor-beta (TGF-beta), and vascular endothelial growth factor (VEGF) [7]. Applying autologous PRF has been found to promote bone and soft-tissue healing around implants. Resonance Frequency Analysis (RFA), introduced by Meredith *et al.* in 1996, offers a non- invasive and practicable method for evaluating implant stability. A vibrating peg attached to the implant detects magnetic pulses, and the resulting signal is converted into an Implant Stability Quotient (ISQ) value in the range of 1 (lowest) to 100 (highest), indicating the level of mechanical stability achieved. Implant stability quotients (ISQs) are the results of measuring implant mobility and stiffness using RFA in the Ostell Mentor Device [8]. Therefore, it is of interest to describe the impact of autologous platelet-rich fibrin on early implant stability and crestal bone changes in maxillary implants positioned in D3 bone during the initial healing phase.

## Methodology:

This study was conducted at the Department of Prosthodontics, Mar Baselios Dental College, Kothamangalam. The Permission and Authorization to perform this research on human participants were obtained from the Ethics Committee of Mar Baselios Dental College. This observational clinical study was conducted in partially edentulous patients aged 18-70 years (both males and females), requiring implant placement in the maxilla. 30 implants in total were placed, 15 each into two groups: Group A (PRF placed in osteotomy site before implant placement) and Group B (no PRF application).Inclusion criteria included D3 bone quality assessed via CBCT, adequate bone volume, good systemic health, and platelet count greater than 200,000/mm^3^. Exclusion criteria included smoking more than 10 cigarettes/day, systemic diseases affecting bone metabolism, recent radiotherapy/chemotherapy, pregnancy, uncontrolled periodontal disease, and parafunctional habits. The study protocol started with a diagnosis appointment. The patients were selected according to the inclusion and exclusion criteria and laboratory investigation results. A thorough clinical evaluation, blood investigation, and a radiographic assessment using cone beam computed tomography (CBCT) were performed to verify the morphological characteristics of the proposed implant site and the positions of surrounding landmarks. Each patient was provided with sufficient information about the procedure and the possible alternatives. Afterwards, a signed consent was obtained from the patient, with their involvement being voluntary. Administration of Local anesthesia followed by making a midcrestal incision with BP blade No. 15 and elevating the full-thickness mucoperiosteal flap. Using the surgical stent, the osteotomy site preparation was initiated with the pilot drill (850 rpm) followed by sequential drilling with increasing diameters and densah burs for Osseo densification. Before implant placement, PRF was placed in the osteotomy site in Group A, whereas Group B received no PRF. Based on Choukroun's protocol, PRF was prepared. Initially, 10 ml of venous blood without an anticoagulant was collected and immediately placed in the REMI centrifuge machine for centrifuging at 2400 rpm for 14 minutes. The PRF clot formed in the middle layer was retrieved with forceps and placed into the osteotomy site before implant placement in Group A, whereas implants in Group B were placed without PRF. The implants were cautiously removed from the vial and positioned at the osteotomy site using a torque wrench and implant driver. Insertion torque and implant stability were measured. Ultimately, healing abutments were fastened, and sutures were placed. Postoperative instructions along with medications were prescribed to the patient. To evaluate the hard tissue changes and implant stability at the specified intervals of time, standardised follow-up exams are planned after 1 week, 1 month and 2 months of implant placement. RVG was applied to measure the loss of crestal bone, and the Osstell beacon was used to check implant stability after 1 week, 1month and 2month of implant placement.

## Results:

The two groups exhibited comparable basal crestal bone levels and primary implant stability (p > 0.05) (Table 1 - see PDF). Group A (PRF) showed stable crestal bone levels with minimal bone loss, while Group B demonstrated significantly greater bone change at 2 months (p < 0.001). The ISQ readings at placement were comparable in both groups (Table 2 - see PDF). Group A exhibited significantly higher ISQ values in both bucco-lingual and mesio-distal orientations at 1 week, 1 month, and 2 months (p < 0.014). The administration of PRF resulted in a positive early response of the crestal bone and enhanced secondary implant stability.

## Discussion:

In the present study, Group A and Group B had similar baseline implant stability and crestal bone levels at implant implantation (p > 0.05). In early healing, implants containing Platelet- Rich Fibrin (PRF) showed greater implant stability quotient (ISQ) values than the control group at several time intervals, including 1 week, 1 month, and 2 months (p < 0.014). In the PRF group, mesio-distal ISQ values increased with time (p = 0.025), while control group values remained consistent. Radiographs revealed bone apposition in the PRF group, while the control group exhibited crestal bone loss by 2 months (p < 0.001). PRF improves secondary implant stability and prevents early crestal bone loss in maxillary implants in D3 bone during biological healing due to the progressive release of bioactive chemicals that promote angiogenesis and osteoblastic activity. According to Öncü and Alaaddinoglu [9], PRF-treated implants showed higher ISQ values during the early healing period which was in agreement with the findings of the present study about enhanced early implant stability and in another study by Öncü and Erbeyoglu [10] leukocyte- and platelet-rich fibrin (L PRF) significantly improved early implant stability and reduced marginal bone loss, especially the first month post placement. Similarly, the results of the current investigation align with those of Tabrizi *et al.* [11] and Kapoor *et al.* [12] who documented markedly enhanced early implant stability with the use of PRF. Similarly, Sunil *et al.* [13] showed that injectable PRF increased ISQ values and decreased marginal bone loss, confirming the advantageous effect of platelet concentrates in early osseointegration. Collectively, these findings support the results of the present study and show the advantage of PRF in the early healing phase. On application of PRF, it stabilizes the blood clot, enhances angiogenesis, promoting early bone formation, thereby preserving implant stability during the critical transition. Whereas, Hussien *et al.* [14] and Khattri *et al.* [15] observed no significant advantage of PRF on implant stability, which may be possibly due to the due to differences in preparation protocols of PRF, implant systems, bone quality, and follow-up periods. The current study's results agree with earlier studies demonstrating the favourable effect of PRF on peri-implant bone preservation. Darestani *et al.* [16] reported substantially decreased marginal bone loss and increased implant stability in posterior maxillary implants when treated with L-PRF. Even in studies by Cheruvu *et al.* [17] and Strauss *et al.* [18] superior crestal bone preservation and reduced bone resorption were observed in implants treated with PRF. Furthermore, studies was also observed supporting the present study such as by Kumar *et al.* [19] and Toffler *et al.* [20] consistently demonstrated reduced crestal bone loss and enhanced early bone formation, supporting the minimal crestal bone changes. Even though, PRF may mitigate crestal bone loss by improving soft tissue healing, its effect on bone preservation appears very inconsistent. Thorat *et al.* [21] and Temmerman *et al.* [22] reported no significant differences in crestal bone levels between PRF and control groups, indicating that PRF may predominantly benefit soft tissue healing rather than bone remodeling, and that its influence on crestal bone is variable and dependent on bone density, surgical technique, and individual biological response.

## Conclusion:

The administration of PRF at osteotomy site significantly enhanced early implant stability and minimized the crestal bone loss in maxilla with D3 bone. PRF serves a simple, autologous, cost-effective adjunct in enhancing the early osseointegration and even may facilitate earlier loading. Long-term studies with larger sample sizes are required to validate these outcomes.
